# Assessment of Potentially Toxic Element Pollution in Surface Soils of the Upper Ohře River Basin

**DOI:** 10.3390/toxics13080644

**Published:** 2025-07-30

**Authors:** Veronika Zemanová, Ladislav Menšík, Edzard Hangen, Bernd Schilling, Lukáš Hlisnikovský, Eva Kunzová

**Affiliations:** 1 Czech Agrifood Research Center, Drnovská 507, 16100 Prague, Czech Republic; ladislav.mensik@carc.cz (L.M.);; 2 Bavarian Environment Agency, Hans-Högn-Strasse 12, 95030 Hof, Germany

**Keywords:** catchment, contamination, Eger, heavy metals/metalloids, toxicity

## Abstract

The soils of river basins are often exposed to contaminants resulting from anthropogenic activities. This research identified 11 potentially toxic elements (PTEs) and assessed pollution indices, ecological risk assessments, and human health risks in the topsoil of the Upper Ohře River Basin (Czech Republic and Germany). Among the PTEs, As, Cd, Cu, Pb, and Zn exhibited considerable variability across the area, with contents exceeding the legislative limits of the Czech Republic, particularly at three locations near coal mining activities. Various indices indicated significant contamination in the river basin (pollution load index > 1, except at one location); however, the level of pollution varied across locations and in relation to the used indices. The ecological risk factor identified As and Cd as the primary pollutants. The potential ecological risk index indicated a strong risk, with two locations showing a highly strong ecological risk. The findings revealed no serious non-carcinogenic or carcinogenic risks to adults, while risks were confirmed for children, with As being the main contributor. This research provides basic information for managing pollution from PTEs and protecting the soils and residents of the Upper Ohře River Basin. In this region, particular attention should be paid towards As and Cd.

## 1. Introduction

Soil, an essential part of the environment, is generally regarded as a vulnerable medium for contamination by potentially toxic elements (PTEs) such as aluminum (Al), arsenic (As), cadmium (Cd), cobalt (Co), chromium (Cr), copper (Cu), iron (Fe), manganese (Mn), nickel (Ni), lead (Pb), and zinc (Zn) due to their bioaccumulation, cumulative toxicity, non-biodegradability, and persistence [1,2,3]. The presence of PTEs in soil is caused by both natural and anthropogenic sources, e.g., soil parent material weathering, soil erosion, mining, smelting, solid waste disposal, traffic emissions, and agricultural practices [4,5]. According to Goren et al. [6], the impact of anthropogenic sources is estimated to surpass that of natural sources, primarily due to increasing industrial activities and urbanization. As a result, research on soil pollution by PTEs predominantly focuses on areas with high levels of human activity [4]. The continuous input of PTEs into the environment and soil poses health risks to humans, plants, and animals [7]. According to Said et al. [8], the environmental impact of PTE is closely linked to its geochemical association in the soil, which is a function of the PTE origin and its reactivity under current or changing environmental conditions. These geochemical associations of PTE have different mobility, and the potential environmental impact of the pollution is enhanced or mitigated through this association and local conditions.

In the soil, the content and distribution of PTEs are influenced by several factors [9], mainly by soil properties such as pH, redox potential, and organic matter content, as well as the interactions among these factors [10,11,12]. In the case of pH, the research showed that a decrease in its value is followed by dissolution processes of minerals and Fe, Mn, and Al hydrous oxides on which PTEs are adsorbed. A decrease in pH of soil affects the availability of nutrients as well as the biological activity of microorganisms [12]. The redox potential is determined by oxidation–reduction reactions, which tend to occur relatively slowly, especially in soil environments [10]. Changes in redox potential affect mineral constituents and soil organic matter as well as regulate the microbial community [13]. Additionally, oxidizing conditions may cause a slight to substantial decrease in soil pH [10]. Furthermore, organic matter significantly affects the soil’s buffering capacity, cation exchange capacity, and the retention of PTEs [10]. In addition to the mentioned factors, PTE mobility is also influenced by the soil type and composition [14]. In general, coarse-grained soils show a lower tendency for PTE adsorption than fine-grained soils.

Accumulation of PTEs in the soil is a global problem that can pose a significant threat to human health [15]. According to these authors, a knowledge gap still exists in the pollution assessment processes. Similarly, Kowalska et al. [16] stated that the total content of PTEs, along with their statistical properties and the correlation between PTE content and soil characteristics, does not provide a complete understanding of the extent of soil pollution. The authors see the key to the effective evaluation of soil contamination by PTEs relying on the application of pollution indices, such as the geo-accumulation index (I_geo_), contamination factor (CF), pollution load index (PLI), etc. [16]. The indices, initially categorized into two groups (individual and complex indices) [17], have also been classified based on their purpose (for example, providing information about individual levels of pollution, the total pollution, the source of PTEs, etc.) and methods of calculation (for example, based on the calculation of geochemical background values, etc.) [16]. Each classified pollution index has strengths as well as weaknesses. Although environmental quality indices share similarities, the differences among them are influenced by various factors. Consequently, the selection of a pollution index is related to different aspects, such as the level of contamination, the origin of PTEs, and the potential ecological risk [16].

This research was focused on the PTE content in topsoil along the Upper Ohře (Eger) River Basin in the Czech Republic and Germany. This river flows from the eastern part of Germany to the Czech Republic and carries pollutants originating from several historical sources in the basin to the Labe (Elbe) River [18]. To date, the pollution history of several locations of the Ohře River, especially sediments, has been documented in previous studies, including those by Matys Grygar et al. [19,20], Fikarová et al. [21], and Majerová et al. [18]. However, the levels of pollution and the assessment of risk using various pollution indices and ecological risk assessment indices in the Upper Ohře River Basin were evaluated for the first time. The purpose of this study was to identify the degree of surface soil pollution by determining PTEs, assess ecological risks, and evaluate human health risks in the study area. Further, this research aims to contribute additional data to previous studies and provide information for pollution control in the studied basin.

## 2. Materials and Methods

### 2.1. Study Area, Sampling, and Laboratory Analysis

**Figure 1:**
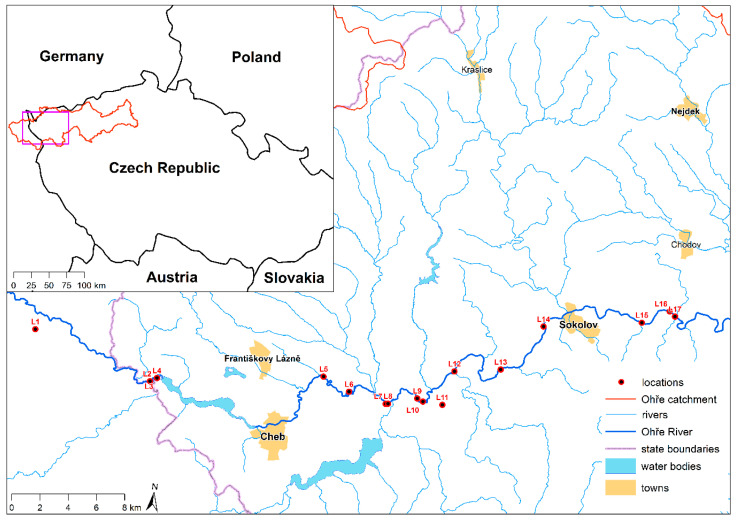
Map illustrating the study area and the specific sampling sites within the Ohře River Basin.

**Figure 2:**
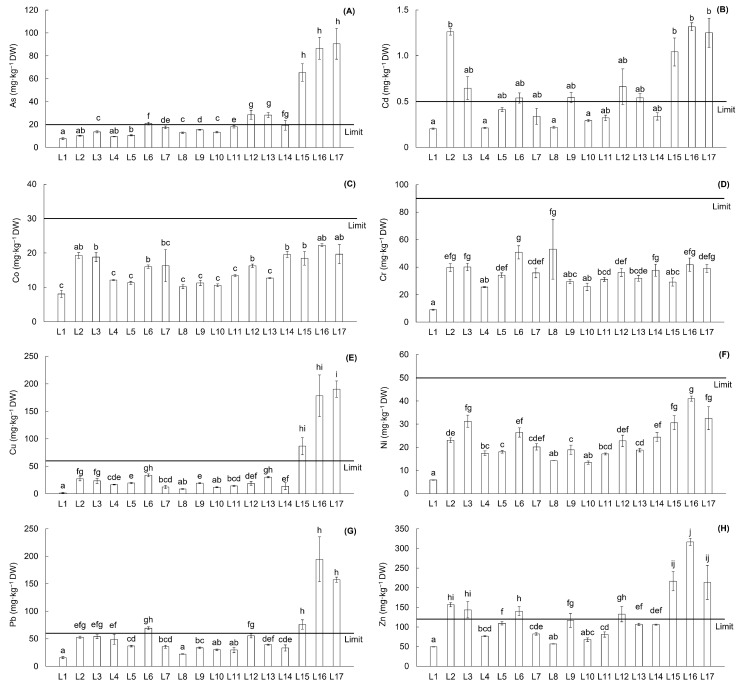
Content (mg·kg^−1^·dry weight^−1^) of potentially toxic elements (PTEs) in soil from sampling locations in the Upper Ohře River Basin with the legislative thresholds of the Czech Republic: As (**A**), Cd (**B**), Co (**C**), Cr (**D**), Cu (**E**), Ni (**F**), Pb (**G**), and Zn (**H**). Letters above the columns indicate differences among locations based on the Kruskal–Wallis one-way ANOVA with the Conover–Iman test (*p* < 0.05; data for As, Cr, Cu, Ni, Pb, and Zn) or the Dunn test (*p* < 0.05; data for Cd), and ANOVA with the Games–Howell test (*p* < 0.05; data for Co). Columns labeled with the same letter do not exhibit statistically significant differences between these locations.

The research was conducted in the Upper Ohře (Eger) River Basin, which encompasses regions in both the Czech Republic and Germany (Figure 1). The Ohře River originates in the Fichtel Mountains near Weißenstadt in eastern Bavaria and extends for 316 km before merging with the Labe River near Terezín in the Czech Republic [19]. The entire catchment area is 5606 km^2^, with potential pollution sources described in detail by Matys Grygar et al. [19,20]. The upper catchment predominantly consists of felsic rocks, and the river features an extensive floodplain [22]. For sampling, 17 locations within the Upper Ohře (Eger) River Basin were selected (Figure 1)—2 locations in Germany (L1 and L2) and 15 locations in the Czech Republic (L3–L17). Each studied location was situated within a floodplain area characterized by Fluvisols as the soil type (Supplementary Table S1). The locations are classified as permanent grasslands or meadows that do not serve a production function and can be utilized as recreational areas. The altitudes of the locations ranged from 390 to 492 m a.s.l. At each location, three separate soil samples were collected from a depth of 0–30 cm, representing the topsoil. Soil samples (approximately 1500 g of soil) were placed in sealed plastic bags and transported to the laboratory. Samples were air-dried, cleared of all foreign matter (rocks, earthworms, germinating plants, roots, etc.), and sieved (<2 mm).

The pH of the samples was measured in water extracts at a 1:5 (*w*/*v*) ratio using an InoLab pH 730 m (WTW Wissenschaftlich-Technische Werkstätten GmbH, Weilheim, Germany). The contents of Ca, K, Mg, and P were determined using an iCAP 7400 Duo ICP-OES spectrophotometer (Thermo Fisher Scientific, Cambridge, UK) following extraction with the Mehlich 3 solution (ratio of 1:10 (*w*/*v*) with solution of 0.2 mol/L CH_3_COOH, 0.25 mol/L NH_4_NO_3_, 0.015 mol/L NH_4_F, 0.013 mol/L HNO_3_, and 0.001 mol/L EDTA) [23]. The contents of PTEs were quantified using the same spectrophotometer after sample digestion in vessels containing 12 mL of aqua regia, performed with an MLS 1200 M microwave digestion system (Milestone Inc., Sorisole, Italy), as described by Zbíral et al. [24]. Blank samples and certified reference material (IRM-5011/I—Luvisol, Central Institute for Supervising and Testing in Agriculture, Brno, Czech Republic, Supplementary Table S2) were used for quality assurance. The variability of results for reference materials was within 10%, and relative standard deviations for sample replicates were ≤7%.

### 2.2. Pollution Indices

#### 2.2.1. Geo-Accumulation Index

The geo-accumulation index (I_geo_) can quantitatively reflect the degree of PTEs pollution in the soil and is calculated as follows [25]:

(1) $$I_{geo}={log}_{2}\left[\frac{C_{n}}{1.5\times B_{n}}\right]$$

where C_n_ is the content of each measured PTE in the sample (mg·kg^−1^·dry weight^−1^); B_n_ is the content of the same PTE in the upper continental crust used as the background values (Al—77,440, As—2, Cd—0.102, Co—11.6, Cu—35, Cr—14.3, Fe—30,890, Mn—527, Ni—18.6, Pb—17, and Zn—237 mg·kg^−1^·dry weight^−1^ [26]); and the value 1.5 is the factor for correction of possible changes in background values in the environment. The classification of I_geo_ reported by Li et al. [27] was used in this study (Table 1).

#### 2.2.2. Contamination Factor and Pollution Load Index

Similarly to I_geo_, the contamination factor (CF) is used to determine the level of soil pollution by PTEs and is calculated as follows [28]:

(2) $$CF=\frac{C_{n}}{B_{n}}$$

where C_n_ is the content of each measured PTE in the sample (mg·kg^−1^·dry weight^−1^), and B_n_ is the background content of the same PTE in the upper continental crust used as the background values (mg·kg^−1^·dry weight^−1^ [26]). The classification of CF reported by Ahmad et al. [28] was used in this study (Table 1).

The pollution load index (PLI) is used to reveal the average amount of soil pollution by all PTEs of interest and is calculated as follows [29]:

(3) $$PLI={(CF}_{1}\times {CF}_{2\;}\ldots \times {CF}_{n}{)}^{1/n}$$

where CF_1_, CF_2_, CF_n_ are CF of PTE 1, 2, …, n, and the PLI values greater than 1 indicate significant contamination [30].

#### 2.2.3. Ecological Risk Factor and Potential Ecological Risk Index

The ecological risk factor (Er) can evaluate the risk associated with each PTE, while the potential ecological risk index (RI) evaluates the cumulative risk of all measured PTEs. Both parameters are calculated as follows [31]:

(4) Er=CF×Tf

(5) RI=∑Er

where CF is the contamination factor of each measured PTE in the sample, and Tf is the toxicity response coefficient of measured PTEs. The Tf values of Al, As, Cd, Co, Cr, Cu, Fe, Mn, Ni, Pb, and Zn are 5, 10, 30, 5, 2, 5, 1, 1, 5, 5, and 1, respectively [32,33,34]. The classification of Er and RI reported by Gong et al. [17] and Kowaslka et al. [16], respectively, was used in this study (Table 1).

### 2.3. Human Health Risk Evaluation

Risk assessment for humans exposed to topsoil contamination by PTEs was determined using the hazard quotient (HQ) and the carcinogenic risk (CR), which reflected non-carcinogenic and carcinogenic effects of PTEs through soil exposure, respectively. In the first step, the health hazard for humans (children and adults) was estimated for oral ingestion by daily intake (ADD_ing_, mg·kg^−1^·day^−1^) and is calculated as follows [35]:

(6) $${ADD}_{ing}=\frac{C_{n}\times IR\times EF\times ED\times 10^{-6}}{BW\times AT}$$

where C_n_ is the content of each measured PTE in the sample (mg·kg^−1^·dry weight^−1^); IR is the soil ingestion rate (200 and 100 mg·day^−1^); EF is the exposure frequency (350 and 250 days·year^−1^); ED is the exposure duration (6 and 26 years); 10^−6^ is the unit conversion factor; BW is the average body weight (15 and 70 kg); and AT is the average time (non-carcinogenic elements: 365 × ED; carcinogenic elements: 2190 and 25,550 days).

In the second step, the HQ of each measured PTE and the hazard index (HI) of all measured PTEs, which can reflect the total potential non-carcinogenic effect, were estimated for oral ingestion and are calculated as follows:

(7) $$HQ=\frac{{ADD}_{ing}}{RfD}$$

(8) HI=∑HQ

where ADD_ing_ is the daily intake by oral ingestion (mg·kg^−1^·day^−1^) and RfD is the reference dose of each measured PTE (mg·kg^−1^·day^−1^). The RfD values of Al, As, Cd, Co, Cu, Cr, Fe, Mn, Ni, Pb, and Zn are 1, 0.0003, 0.001, 0.0003, 0.04, 0.003, 0.7, 0.14, 0.02, 0.0035, and 0.3, respectively [36,37,38]. The HQ and HI values less than 1 indicate the non-harmful risk of exposure, while the HQ and HI greater than 1 indicate the existence of an adverse, non-carcinogenic health risk of exposure [39].

The potential carcinogenic risks were assessed only for As, Cd, Cr, Ni, and Pb, which are considered carcinogenic elements. The risk was estimated individually by CR and as a cumulative effect by total carcinogenic risk (TCR). Both parameters were estimated for oral ingestion and are calculated as follows [38]:

(9) $$CR=\frac{{ADD}_{ing}}{SF}$$

(10) TCR=∑CR

where ADD_ing_ is the daily intake by oral ingestion (mg·kg^−1^·day^−1^) and SF is the cancer slope factor (mg·kg^−1^·day^−1^) of each carcinogenic element. The SF values of As, Cd, Cr, Ni, and Pb are 1.5, 0.38, 0.001, 0.5, 1.7, and 0.0085, respectively [34,36,38]. The CR and TCR values of 1 × 10^–6^ to 1 × 10^–4^ indicate an acceptable risk level, while CR and TCR values exceeding 1 × 10^−4^ indicate a high-risk level of cancer to human health [40].

### 2.4. Statistical Analyses

Normality of the data was checked using the Shapiro–Wilk test. The effect of locations, due to the non-normally distributed data, was evaluated with the Kruskal–Wallis one-way ANOVA, followed by the Conover–Iman post hoc test for As, Cr, Cu, Fe, Mn, Ni, Pb, Zn, PLI, RI, HI, and TCR, and the Dunn test for Cd. Due to the normally distributed data for Al and Co, a one-way ANOVA was applied, followed by the Games–Howell post hoc test to account for the heterogeneity of variances. All analyses were conducted using Statistica 14.0 (Tibco Software, Palo Alto, CA, USA) and XLStat version 2023.1.3 software (Lumivero, Burlington, MA, USA). Figures were created by Excel—Microsoft Office LTSC Standard 2021 (Microsoft s.r.o., Prague, Czech Republic).

## 3. Results

**Figure 3:**
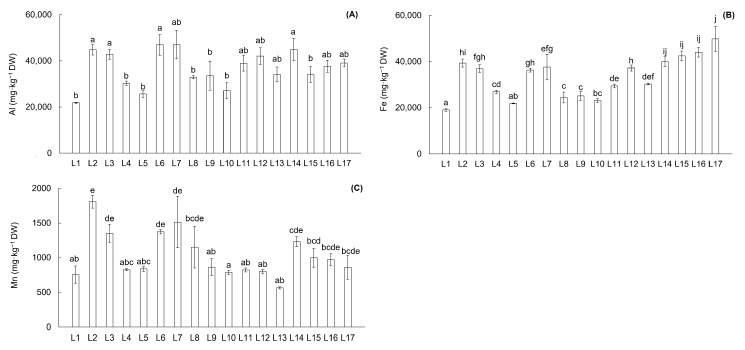
Content (mg·kg^−1^·dry weight^−1^) of potentially toxic elements (PTEs) in soil from sampling locations in the Upper Ohře River Basin without determined legislative thresholds of the Czech Republic: Al (**A**), Fe (**B**), and Mn (**C**). Letters above the columns indicate differences among locations based on the Kruskal–Wallis one-way ANOVA with the Conover–Iman test (*p* < 0.05; data for Fe and Mn) and ANOVA with the Games–Howell test (*p* < 0.05; data for Al). Columns labeled with the same letter do not exhibit statistically significant differences between these locations.

In the soils of the Upper Ohře River Basin locations, 11 elements categorized as PTEs were determined—Al, As, Cd, Co, Cr, Cu, Fe, Mn, Ni, Pb, and Zn. The average contents of these PTEs in the soils of the river basin are presented in Table 2, while the PTE contents in soils of individual locations are shown in Figure 2 and Figure 3. The mean content of PTEs in soil samples showed the following trend: Al > Fe > Mn > Zn > Pb > Cu > Cr > As > Ni > Co > Cd (Table 2). The contents of As, Cd, Cu, Pb, and Zn displayed considerable variability across the Upper Ohře River Basin. These elements, together with Co, Cr, and Ni, are monitored in the soils of the Czech Republic and have legislative thresholds—prevention values defined as the content of the individual element determined in aqua regia soil extract (No. 153/2016). Other determined elements—Al, Fe, and Mn—are without legislative thresholds in the Czech Republic. According to the legislative prevention limits for PTE contents in soils, the average contents of As, Cd, and Zn exceeded the reference values (Table 2). At the individual locations, elevated contents surpassing the regulatory reference values were observed for As, Cd, Cu, Pb, and Zn (Figure 2). Specifically, elevated contents were observed at location L2 for Cd (2.5 and 1.3 times higher, respectively) and Zn (1.3 and 1.2 times higher, respectively). Location L6 showed lower elevation of As, Cd, Pb, and Zn (1.04, 1.1, 1.2, and 1.2 times higher, respectively) compared to the reference values. Location L9 showed weak elevation of Cd—1.1 times higher. Similarly, low elevation of As (1.4 times higher), Cd (1.3 times higher), and Zn (1.1 times higher) compared to the reference value was observed at location L12. Furthermore, location L13 demonstrated 1.4 times higher As content and 1.1 times higher Cd content compared to the reference values. On the other hand, locations L15, L16, and L17 showed higher elevations of As (3.3, 4.3, and 4.5 times higher, respectively), Cd (2.1, 2.6, and 2.5 times higher, respectively), Cu (1.4, 3.0, and 3.2 times higher, respectively), Pb (1.3, 3.2, and 2.6 times higher, respectively), and Zn (1.8, 2.6, and 1.8 times higher, respectively) compared to the reference values. The locations with elevated content of PTEs were characterized as highly acidic soils (L12), acidic soils (L2, L6, L9, L13, and L17), and slightly acidic soils (L3, L15, and L16).

Selected soil contamination indicators, such as the geo-accumulation index (I_geo_), contamination factor (CF), pollution load index (PLI), ecological risk factor (Er), and potential ecological risk index (RI), were used to assess the level of soil quality and pollution in the river basin. These indicators help to determine the potential risk and toxicity to the environment due to PTEs in the soil.

The determination of I_geo_ and CF was used to evaluate the soil quality and the pollution of the river basin by individual PTEs. The content of PTEs from the upper continental crust was used for both parameters. The values of I_geo_ showed that the locations of the river basin can be overall described as non-contaminated by Al, Fe, and Zn. In the case of other PTEs, the locations of the river basin can be categorized as non-contaminated to highly contaminated (Supplementary Table S3). Higher contamination was calculated for As, Cd, and Pb, especially at locations L15, L16, and L17 of the river basin.

On the other hand, the CF is classified as low contamination when values are <1 (Table 1). Based on CF classification, most studied locations in the river basin are moderately contaminated by individual PTEs (Supplementary Table S4). In the case of As, Cd, Cr, Mn, and Pb, some locations are classified as considerably contaminated. Additionally, very high contamination was calculated for As (L3 and L6–L17), Cd (L2, L3, L12, and L15–L17), and Pb (L16 and L17). The values indicate especially high CF for As at locations of river basin L15, L16, and L17 (Supplementary Table S4).

**Figure 4:**
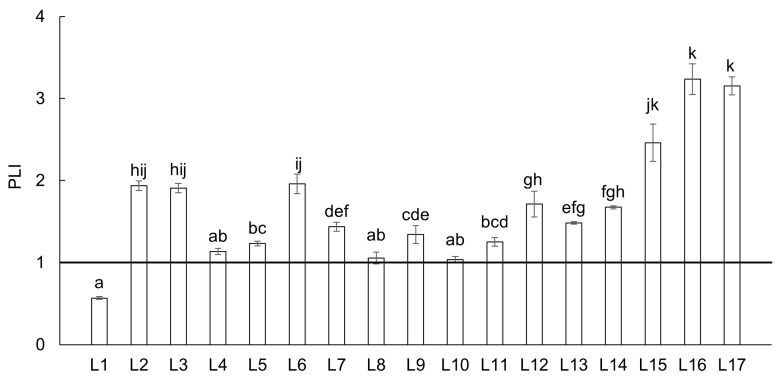
Pollution load index (PLI) in soil from all sampling locations in the Upper Ohře River Basin. Values of PLI < 1 are for unpolluted soils. Letters above the columns indicate significant differences among locations based on the Kruskal–Wallis one-way ANOVA with the Conover–Iman test (*p* < 0.05). Columns labeled with the same letter do not exhibit statistically significant differences between these locations.

Both I_geo_ and CF characterize only the individual PTEs at specific locations of the river basin; therefore, the PLI was calculated to determine the combined effect of all PTEs at the studied locations in the river basin (Figure 4). A PLI value of 1 indicates a baseline level of pollution, while values greater than 1 indicate polluted soils. According to this classification, the soil quality of all locations in the Upper Ohře River Basin, except L1, was affected and can be described as polluted, with locations L2, L3, L6, L15, L16, and L17 being particularly impacted (Figure 4). The PLI values for these localities were 1.9 to 3.2 times higher compared to the baseline values.

The potential ecological risk (Er) of individual PTEs and the potential ecological risk index (RI), which is used to depict the cumulative impact of all studied PTEs, were used to evaluate the environmental risks of PTEs in the studied locations of the Upper Ohře River Basin.

Based on the Er classification (Table 1), all PTEs at all locations in the river basin, except As, Cd, and Pb, can be described as low risk (Supplementary Table S5). For As, location L1 is categorized as low risk, locations L2–L5, L8, and L10 as moderate risk, locations L6, L7, L9, and L11–L14 as considerable risk, and locations L15–L17 as extreme risk. For Cd, all locations—except L1, L4, and L8 (moderate risk); L12 and L15 (high risk); and L16 and L17 (extreme risk)—can be categorized as considerable risk. For Pb, all locations, except L16 and L17 (moderate risk), can be described as low risk. The results of Er indicate a high contribution of As and Cd to the environmental risk of the studied locations (Supplementary Table S5).

**Figure 5:**
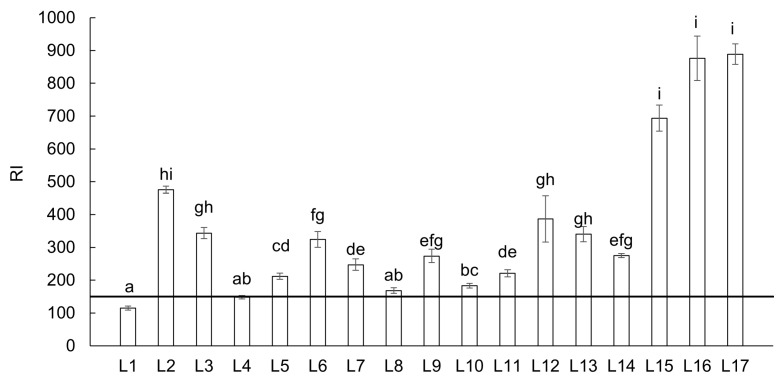
Potential ecological risk index (RI) from all sampling locations in the Upper Ohře River Basin. A value of RI < 150 indicates low potential ecological risk. Letters above the columns indicate significant differences among locations based on the Kruskal–Wallis one-way ANOVA with the Conover–Iman test (*p* < 0.05). Columns labeled with the same letter do not exhibit statistically significant differences between these locations.

The RI depicts the potential ecological threat posed by all PTEs in the environment. According to the RI classification (Table 1), most localities fall into a strong ecological risk level (Figure 5). Three localities (L1, L4 and L8) exhibited a moderate ecological risk level. Another three locations (L2, L12 and L15) had a very strong ecological risk level, while only two locations (L16 and L17) had a highly strong potential ecological risk.

The potential health risk of individual contaminants like PTEs is typically assessed based on the estimated risk levels and categorized as either non-carcinogenic or carcinogenic. The determination of hazard quotient (HQ), hazard index (HI), carcinogenic risk (CR), and total carcinogenic risk (TCR) was used to evaluate the degree of PTEs pollution of the Upper Ohře River Basin and possible health risks for children and adults.

**Figure 6:**
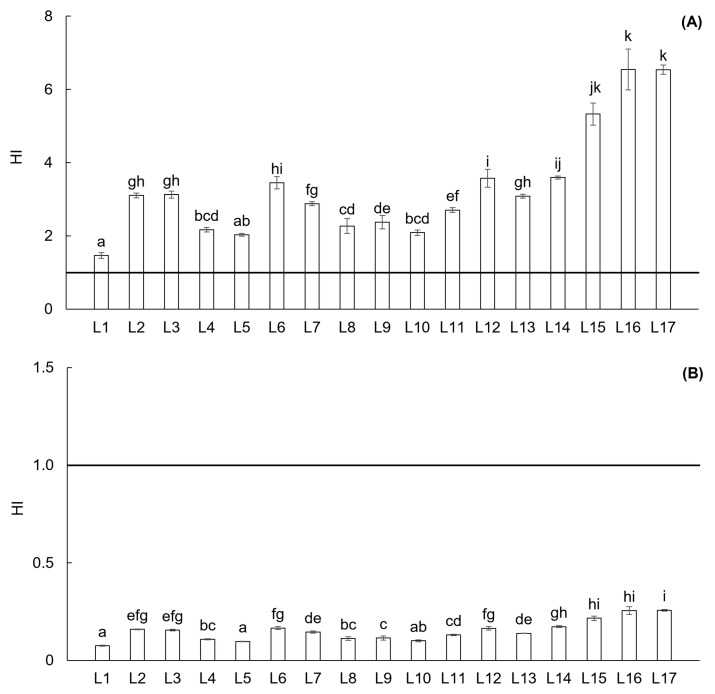
Hazard index (HI) from all sampling locations in the Upper Ohře River Basin for children (**A**) and adults (**B**). A value of HI < 1 indicates a non-harmful risk of exposure. Letters above the columns indicate significant differences among locations based on the Kruskal–Wallis one-way ANOVA with the Conover–Iman test (*p* < 0.05). Columns labeled with the same letter do not exhibit statistically significant differences between these locations.

The HQ and HI of oral ingestion evaluate the non-carcinogenic health impact due to the exposure to individual PTEs or the combination of all PTEs, respectively. An HQ and HI value of 1 indicates a baseline level of harmful health impact, while values exceeding 1 suggest a possible non-carcinogenic risk for both children and adults. Based on the HQ classification, possible non-carcinogenic impact of oral ingestion for children was determined for locations L12, L13, and L15–L17 (Supplementary Table S6). The HQ values for these locations were 1.2 to 3.4 times higher compared to the baseline. On the other hand, all locations in the Upper Ohře River Basin had low risk of exposure for adults—HQ under 1 (Supplementary Table S7). The results of HI indicate non-carcinogenic impact by oral ingestion for children at all locations, especially at L15–L17 with values 5.3 to 6.5 times higher compared to the baseline (Figure 6A). Opposite results—low impact of exposure to the combination of all PTEs—were determined for adults (Figure 6B).

**Figure 7:**
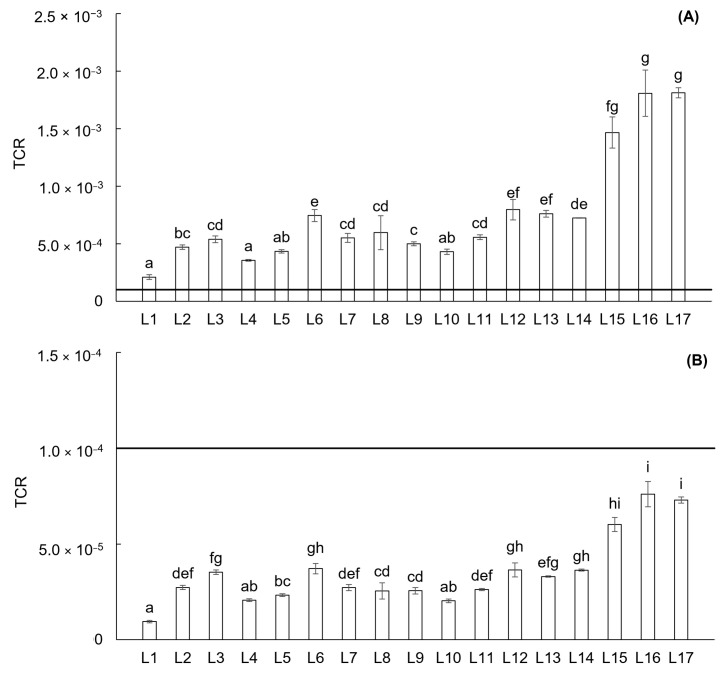
Total carcinogenic risk (TCR) from all sampling locations in the Upper Ohře River Basin for children (**A**) and adults (**B**). A value of TCR exceeding 1 × 10^−4^ indicates a high-risk level of cancer to human health. Letters above the columns indicate significant differences among locations based on the Kruskal–Wallis one-way ANOVA with the Conover–Iman test (*p* < 0.05). Columns labeled with the same letter do not exhibit statistically significant differences between these locations.

The CR and TCR are considered as the possibility of an individual developing any type of cancer in their whole lifetime due to exposure to carcinogenic hazards [41]. Carcinogenic health risks for children and adults were assessed individually for As, Cd, Cr, Ni, and Pb by CR and for combinations of these elements by TCR. CR and TCR values exceeding 1 × 10^−4^ indicate a high-risk level of cancer to human health. Results of CR for the children indicate carcinogenic risk of As at all locations (Supplementary Table S8). In addition, carcinogenic risk for children was calculated for Cr at locations L2–L17. On the other hand, all locations in the Upper Ohře River Basin are without carcinogenic risks for adults (Supplementary Table S9). In the case of As, Cd, Cr, Ni, and Pb combined risk, the results of TCR indicate carcinogenic risk for children at all locations, especially L15–L17 (Figure 7A). The values of TCR for adults indicate for all locations no carcinogenic risk (Figure 7B).

## 4. Discussion

### 4.1. Content of Potentially Toxic Elements in the Soil of the Upper Ohře River Basin

The soil of all sampling locations was classified as Fluvisols, which, according to Vácha et al. [42], generally contain PTEs predominantly due to anthropogenic factors in the Czech Republic. In the soils of the Upper Ohře River Basin, the highest mean content showed Al, Fe, and Mn that are not legislatively regulated in the Czech Republic. These elements are relatively abundant in soils and are widely distributed in nature [43,44]. According to Reimann et al. [45], in European agricultural soils, Al content fluctuates from 329 to 65,090 mg·kg^−1^ soil (median 10,993 mg·kg^−1^), Fe content ranges from 392 to 133,926 mg·kg^−1^ (median 17,200 mg·kg^−1^), and Mn content varies from 2 to 14,969 mg·kg^−1^ (median 445 mg·kg^−1^). The contents of these elements at all locations in the Upper Ohře River Basin reached values within the mentioned ranges. Furthermore, for Al and Fe, all locations can be overall classified as non-contaminated based on the results of I_geo_, which assessed the pollution of the soils of the Upper Ohře River Basin for individual PTEs. Higher variability in pollution levels was observed for Mn, with locations categorized as non-contaminated to low contaminated. The pollution levels of individual PTEs were also assessed using the CF. According to CF, the soils of the Upper Ohře River Basin exhibit low levels of contamination with Al and low to moderate levels with Fe, while Mn contamination ranges from moderate to considerable.

The mean content of other PTEs that are legislatively regulated in the Czech Republic declined in the following order: Zn > Pb > Cu > Cr > As > Ni > Co > Cd. Similar results, with a decline in the order of Zn > Pb > Cu > Cd, were observed by Matys Grygar et al. [19] for sediments in the Upper Ohře River Basin. Results obtained from topsoil samples in the Upper Ohře River Basin by Skála et al. [46] showed a similar trend, with a decline in the order of Zn > Ni > Cu > Pb > As > Cd. The natural contents of PTEs in floodplain sediments in the upper reaches of the Ohře River are comparable to the mean contents of the upper crust [19]. Research on the sediments of the Ohře River system confirmed that contamination was mainly by As, Cu, Pb, tin (Sn), and Zn [18,20,47]. Additionally, research on topsoil samples confirmed that contamination was mainly by Cd, As, and Cu in the upper part of the basin [46]. Our results from the Upper Ohře River Basin showed considerable variability in the contents of As, Cd, Cu, Pb, and Zn across the river basin. In the case of average PTE contents in the Upper Ohře River Basin, the prevention limits set by Czech legislation were exceeded for As, Cd, and Zn, while individual locations exceeded these limits for As, Cd, Cu, Pb, and Zn. All these elements surpassed the prevention limits at locations L15, L16, and L17, which are located in the Sokolov area, where coal mining activities have been conducted since the 19th century [20]. Furthermore, the As contents at these three locations exceeded another legislative limit—the indication limit for human health protection (40 mg·kg^−1^ dry weight).

The pollution of soils in the Upper Ohře River Basin was assessed using the I_geo_ and CF for individual PTEs, as well as the PLI for the combined effects of all PTEs. These parameters have also been used by Hoque et al. [3], Jain et al. [48], and Rivera-Hernández et al. [49] to evaluate the intensity of PTE content in river basin soils. According to the I_geo_ results, the locations within the Upper Ohře River Basin can be classified as non-contaminated with respect to Zn. However, for other PTEs, the locations range from non-contaminated to highly contaminated. The findings indicate higher contamination for As, Cd, and Pb, especially at locations L15 to L17. The CF results reveal that most studied locations are moderately contaminated. Additionally, some locations are classified as considerably contaminated by As, Cd, Cr, Mn, and Pb, while others are categorized as very highly contaminated by As, Cd, and Pb.

The results of PLI for the combined impact of PTEs classified all locations in the Upper Ohře River Basin, except for L1, as polluted, indicating affected soil quality. These results agree with Matys Grygar et al. [19], who provided an overview of sediment contamination sources linked to both ancient (Pb, Ag, and Cu ore mining from the 14th to 19th century) and modern (uranium ore mining between 1952 and 1965; coal mining in the Sokolov basin since the 19th century) mining and processing in the Ohře River Basin. Additionally, Skála et al. [46] reported contributions of ancient ore mining and processing in the broader region of the Ohře River catchment. In the case of locations L15, L16, and L17, the source of contamination can be attributed to coal mining activities in the Sokolov basin [19,20]. Furthermore, the Ohře floodplain has also been impacted by the enrichment of As, Pb, Cu, and Zn from sediments of tributaries [19]. In relation to this phenomenon, the source of contamination in the case of L2 and L3 can be attributed to the Röslau River, which transported pollution (mainly Hg) from an abandoned factory in Marktredwitz, Germany, that was active from 1788 to 1985 [19].

### 4.2. Ecological Risk Assessment

The RI, which is used to evaluate the sensitivity of diverse biological communities and the detrimental effects on the environment and ecosystems [50], was obtained by calculating the Er. The calculation of Er is a crucial method for evaluating the risk of PTEs, as it considers both the content and the toxicity response coefficient of these elements [51]. The results of Er for all individual PTEs, except for As, Cd, and Pb at certain locations, indicated a low risk across all locations in the river basin. Conversely, the Er results for As revealed moderate, considerable, and very high risk at 41.2, 35.3, and 17.6% of locations in the river basin, respectively. Among the locations in the Upper Ohře River Basin, the highest risk was associated with Cd. Similarly, other researchers have reported a high ecological risk for Cd due to its content and higher toxicity coefficient [52,53]. In the Upper Ohře River Basin, a very high risk for Cd was identified at three locations, while considerable and high risks were observed at 47.1 and 17.6% of locations, respectively. The significant contribution of Cd, along with As and Cu, in the Ohře River Basin, may be attributed, as noted by Skála et al. [46], to the spatial interaction of various pollution sources in the region, including long-term airborne pollution from fossil fuel combustion and the chemical industry, as well as geochemical anomalies in metallogenic zones [54].

In the case of the combined effects of PTEs, RI values indicated that only two locations (L16 and L17) exhibit a highly strong ecological risk level. Strong ecological risks were observed in 52.9% of the locations, while the remaining locations were classified as having moderate (17.6%) and very strong (17.6%) ecological risk. These results are in accordance with Er’s results and confirmed Cd and As as the main contributors to the RI. Similar results have been reported by Skála et al. [46] in the topsoils of the upper part of the Ohře River Basin, as well as in studies from other countries, including Mugoša et al. [52], Zhang et al. [53], and Pan et al. [55]. Additionally, the study by Liu et al. [56] presented Cd together with As as elements posing a very high ecological risk in a river basin impacted by historical mining and industrial activities.

### 4.3. Potential Human Health Risk

The potential non-carcinogenic health risks associated with the ingestion of soil particles for both children and adults, which is a significant route of exposure to PTEs [27,57], were assessed using the HQ and HI. For individual PTEs, only As, a hazardous metalloid that typically ranked as the 12th most abundant element in the human body [58], posed a risk at five locations within the Upper Ohře River Basin for children. In contrast, exposure to individual PTEs for adults presented a low risk across all locations. Similarly, when considering the combined impact of all PTEs (HI), all locations in the Upper Ohře River Basin posed a low risk to adults; however, the risk was confirmed for children. According to Pan et al. [57], the higher HI values for children can be attributed to their increased susceptibility to a given dose of PTEs, as they are likely to inadvertently ingest significant quantities of soil due to pica behavior, hand- or thumb-sucking, and outdoor play activities. Therefore, it is recommended to implement protective measures for children in contaminated locations [27]. Additionally, the findings of Yan et al. [35] demonstrated that children are more sensitive than adults due to their unique exposure characteristics. These authors also identified ingestion as the primary exposure risk, with As and Ni posing non-carcinogenic risks for children in the studied Chinese provinces.

Due to their associated hazards, selected PTEs (As, Cd, Cr, Ni, and Pb) were evaluated for their carcinogenic impact using CR and TCR assessments. For individual PTEs, the carcinogenic impact (CR) for children indicated that As and Cr posed a risk at all locations within the Upper Ohře River Basin, except for location L1 concerning Cr. In contrast, exposure to individual PTEs did not present a carcinogenic risk for adults at any location. Similarly, evaluation of the combined impact from all PTEs (TCR) revealed that none of the locations in the Upper Ohře River Basin posed a carcinogenic risk to adults. However, a carcinogenic risk was confirmed for children at all locations. These results align with those of Pan et al. [57], who reported that children are more susceptible to carcinogenic risks in their daily lives due to unintentional ingestion pathways.

## 5. Conclusions

In the soils of the Upper Ohře River Basin, the most abundant PTEs were Al, Fe, and Mn. The results of the I_geo_ classified the soils as non-contaminated by Al and Fe, while indicating a non-contaminated to low-contaminated status for Mn. The soil samples were classified by CF calculation as low contaminated by Al, low to moderately contaminated by Fe, and low to considerably contaminated by Mn. The eight other identified PTEs, which are legislatively regulated in the Czech Republic, followed this ranking: Zn > Pb > Cu > Cr > As > Ni > Co > Cd. Among these PTEs, As, Cd, Cu, Pb, and Zn surpassed the legislative limits established by the Czech Republic, with their contents exhibiting considerable variability across the studied area. Despite the elevated content of Zn, the I_geo_ classified the soils of the Upper Ohře River Basin as non-contaminated on an overall level. Contamination levels for As, Cd, Cu, and Pb were classified as low to high. Similarly, the CF calculation indicated that the soil samples were low to moderately contaminated, with some locations classified as having considerable to very high contamination. The results of PLI assessed all locations, except for one location (L1), as polluted, indicating affected soil quality. Ecological risk was evaluated by Er and RI calculations, which identified a low risk for the majority of PTEs, except for As, Cd, and Pb. Specifically, Cd and As were identified as the primary pollutants across the studied locations in the Upper Ohře River Basin. Based on the RI results, nine locations exhibited strong ecological risk, while two locations showed highly strong ecological risk. Regarding ingestion risk for human health, the HQ and HI results indicated a low non-carcinogenic risk to adults. Similarly, the CR and TCR assessments showed no carcinogenic risk to adults. In contrast, these four factors indicated non-carcinogenic as well as carcinogenic risks to children, with As being the primary contributor. The research provides valuable information on soil contamination in the Upper Ohře River Basin, which can help in the development of effective regulations and policies designed to protect the health of residents, particularly children, both within and around the study area.

## Figures and Tables

**Table 1:** Pollution indices classification.

I_geo_	Level	CF	Level
I_geo_ < 0	Non-contamination	CF < 1	Low contamination
0 < I_geo_ < 1	Slight contamination	1 ≤ CF < 3	Moderate contamination
1 ≤ I_geo_ < 2	Low contamination	3 ≤ CF < 6	Considerable contamination
2 ≤ I_geo_ < 3	Moderate contamination	CF ≥ 6	Very high contamination
3 ≤ I_geo_ < 4	Heavy contamination		
4 ≤ I_geo_ < 5	High contamination		
I_geo_ ≥ 5	Extreme contamination		
**Er**	**Level**	**RI**	**Level**
Er < 40	Low potential ecological risk	RI < 90	Low potential ecological risk
40 ≤ Er < 80	Moderate potential ecological risk	90 ≤ RI < 180	Moderate potential ecological risk
80 ≤ Er < 160	Considerable potential ecological risk	180 ≤ RI < 360	Strong potential ecological risk
160 ≤ Er < 320	High potential ecological risk	360 ≤ RI < 720	Very strong potential ecological risk
Er ≥ 320	Very high potential ecological risk	RI ≥ 720	Highly strong potential ecological risk

**Table 2:** Content (mg·kg^−1^·dry weight^−1^) of potentially toxic elements (PTEs) in soil from all sampling locations in the Upper Ohře River Basin and the legislative thresholds of the Czech Republic.

	Min	Max	Mean	Median	SD	CV	Limit Value ^a^
Al	21,505.34	53,222.06	36,228.40	36,944.41	7974.53	22.01	-
As	6.57	89.14	26.08	17.38	22.15	84.94	20
Cd	0.20	1.42	0.59	0.48	0.36	59.99	0.5
Co	6.98	22.68	14.71	13.52	4.00	27.23	30
Cr	8.54	74.93	34.30	33.03	10.25	29.88	90
Cu	0.93	185.91	36.69	19.55	43.86	119.52	60
Fe	18,658.60	46,082.50	32,076.07	30,654.11	8064.81	25.14	-
Mn	555.63	1875.64	1016.50	863.13	318.44	31.33	-
Ni	5.78	42.15	21.99	19.67	7.98	36.29	50
Pb	14.42	177.69	53.28	39.07	35.71	67.01	60
Zn	49.16	321.39	128.51	110.74	64.54	50.22	120

^a^ Legislative limits for soil (Act No. 153/2016).

## Data Availability

The data presented in this research are available in this article and Supplementary Materials.

## Supplementary Materials

The following supporting information can be downloaded at: https://www.mdpi.com/article/10.3390/toxics13080644/s1. Table S1: Basic properties of soil from all sampling locations in the Upper Ohře River Basin; Table S2: Reference material and quality assurance; Table S3: Geo-accumulation index (I_geo_) of potentially toxic elements in soil from sampling locations in the Upper Ohře River Basin; Table S4: Contamination factor (CF) of potentially toxic elements in soil from all sampling locations in the Upper Ohře River Basin; Table S5: Ecological risk factor (Er) of potentially toxic elements from all sampling locations in the Upper Ohře River Basin; Table S6: Hazard quotient (HQ) of potentially toxic elements for children from all sampling locations in the Upper Ohře River Basin. A value of HQ < 1 indicates a low risk of exposure; Table S7: Hazard quotient (HQ) of potentially toxic elements for adults from all sampling locations in the Upper Ohře River Basin. A value of HQ < 1 indicates a low risk of exposure; Table S8: Carcinogenic risk (CR) of potentially toxic elements for children from all sampling locations in the Upper Ohře River Basin. A value of CR exceeding 1 × 10^−4^ indicates a high-risk level of cancer to human health; Table S9: Carcinogenic risk (CR) of potentially toxic elements for adults from all sampling locations in the Upper Ohře River Basin. A value of CR exceeding 1 × 10^−4^ indicates a high-risk level of cancer to human health.
