# Supplementary material for: Assessment of Potentially Toxic Element Pollution in Surface Soils of the Upper Ohře River Basin
Source: Toxics. 2025 Jul 30;13(8):644. doi: 10.3390/toxics13080644 (PMC12390311; doi:10.3390/toxics13080644)
Supplement: Supplementary file 1 [file toxics-13-00644-s001.zip › Supplementary Table S1.pdf]

Table S1 Basic properties of soil from all sampling locations in the Upper Ohře River Basin

Location		pH (H_2O)	Ca	Mg	P	K	Na
					(mg·kg^{-1})		
1	Leupoldshammer	5.1	527.06	44.01	15.70	48.11	165.72
2	Fischern	5.0	2317.43	151.47	46.53	79.22	364.49
3	Pomezí nad Ohří	5.7	2165.78	233.37	38.87	83.58	317.87
4	Cheb	5.5	1208.24	99.44	41.44	119.44	169.03
5	Chocovice	6.5	2167.45	225.30	62.20	59.29	227.10
6	Vokov	5.3	1717.08	157.10	48.06	97.27	355.04
7	Odrava/1	7.8	13073.91	133.90	23.56	67.32	356.63
8	Odrava/2	7.1	3392.91	108.62	123.81	100.79	283.58
9	Chotíkov/1	5.1	1471.93	160.94	55.56	81.52	286.95
10	Chotíkov/2	7.4	3854.24	129.01	18.22	42.44	200.87
11	Dolní Pochlovice/1	6.2	2501.82	197.02	56.43	59.17	260.47
12	Dolní Pochlovice/2	4.3	842.50	128.47	29.55	192.10	255.50
13	Šabina	5.3	1569.15	114.54	16.76	92.24	231.66
14	Citice	5.5	1919.33	174.40	16.07	93.38	281.39
15	Nové Sedlo	5.6	1747.11	217.43	46.50	135.42	436.42
16	Loket/1	5.6	2015.16	248.05	34.99	227.00	403.92
17	Loket/2	4.8	1411.56	172.24	34.69	221.02	414.60
