# Supplementary material for: Assessment of Potentially Toxic Element Pollution in Surface Soils of the Upper Ohře River Basin
Source: Toxics. 2025 Jul 30;13(8):644. doi: 10.3390/toxics13080644 (PMC12390311; doi:10.3390/toxics13080644)
Supplement: Supplementary file 1 [file toxics-13-00644-s001.zip › Supplementary Table S2.pdf]

Table S2 Reference material and quality assurance.

	IRM-5011/I (Luvisol)		Recovery rates (%)	Instrument detection limit (mg/kg, iCAP 7400 Duo)
	**Certified**	**Determined**		
**Al**	920.90 ± 49.02	972.47 ± 48.62	105.6	0.005
**As**	6.92 ± 0.67	7.00 ± 0.16	101.2	0.2
**Cd**	0.14 ± 0.04	0.13 ± 0.01	94.4	0.007
**Co**	6.22 ± 0.47	6.04 ± 0.30	97.2	0.025
**Cu**	7.01 ± 0.41	7.34 ± 0.33	104.6	0.05
**Cr**	25.22 ± 3.02	26.12 ± 0.43	103.6	0.02
**Fe**	1019.33 ± 145.23	1048.28 ± 68.14	102.8	0.03
**Mn**	232.33 ± 33.39	215.09 ± 14.30	92.6	0.004
**Ni**	11.92 ± 0.89	12.16 ± 0.36	102.0	0.03
**Pb**	18.57 ± 2.03	17.76 ± 1.03	95.6	0.1
**Zn**	25.44 ± 2.53	24.02 ± 1.46	94.4	0.01
