# Supplementary material for: Assessment of Potentially Toxic Element Pollution in Surface Soils of the Upper Ohře River Basin
Source: Toxics. 2025 Jul 30;13(8):644. doi: 10.3390/toxics13080644 (PMC12390311; doi:10.3390/toxics13080644)
Supplement: Supplementary file 1 [file toxics-13-00644-s001.zip › Supplementary Table S3.pdf]

Table S3 Geo-accumulation index (I_{geo}) of potentially toxic elements in soil from sampling locations in the Upper Ohře River Basin

	I_{geo}										
	**Al**	**As**	**Cd**	**Co**	**Cr**	**Cu**	**Fe**	**Mn**	**Ni**	**Pb**	**Zn**
**L1**	-2.41	1.34	0.41	-1.12	-5.21	-1.25	-1.28	-0.08	-2.25	-0.72	-2.84
**L2**	-1.38	1.77	3.04	0.15	-0.95	0.88	-0.24	1.19	-0.28	1.05	-1.18
**L3**	-1.41	2.12	2.30	0.15	-0.94	0.95	-0.35	0.81	0.24	1.08	-1.14
**L4**	-1.94	1.64	0.46	-0.52	-1.65	0.25	-0.78	0.06	-0.68	0.93	-2.21
**L5**	-2.18	1.81	1.43	-0.62	-1.43	0.67	-1.09	0.08	-0.63	0.53	-1.70
**L6**	-1.31	2.80	1.81	-0.12	-0.65	1.24	-0.35	0.80	-0.08	1.44	-1.35
**L7**	-1.47	2.50	1.43	-0.37	-1.81	0.62	-0.49	1.12	-0.55	0.39	-2.07
**L8**	-1.82	2.10	0.52	-0.77	-2.60	1.22	-0.93	0.51	-0.97	-0.20	-2.65
**L9**	-1.81	2.37	1.82	-0.63	-1.45	0.46	-0.89	0.12	-0.57	0.39	-1.63
**L10**	-2.11	2.15	0.93	-0.72	-2.15	0.25	-1.01	-0.01	-1.06	0.24	-2.40
**L11**	-1.58	2.59	1.06	-0.38	-1.87	0.54	-0.66	0.06	-0.71	0.20	-2.14
**L12**	-1.47	3.24	2.06	-0.10	-1.50	0.75	-0.32	0.02	-0.30	1.12	-1.44
**L13**	-1.77	3.24	1.81	-0.45	-0.80	0.57	-0.62	-0.49	-0.57	0.62	-1.73
**L14**	-1.34	2.95	1.29	0.14	-1.41	0.94	-0.15	0.57	-0.10	0.59	-1.73
**L15**	-1.78	4.44	2.75	0.08	0.70	0.44	-0.13	0.32	0.13	1.57	-0.72
**L16**	-1.59	4.72	3.08	0.38	1.46	0.83	-0.11	0.41	0.54	2.63	-0.17
**L17**	-1.61	4.74	3.12	0.28	1.78	0.78	-0.03	0.33	0.34	2.60	-0.56
