# Supplementary material for: Assessment of Potentially Toxic Element Pollution in Surface Soils of the Upper Ohře River Basin
Source: Toxics. 2025 Jul 30;13(8):644. doi: 10.3390/toxics13080644 (PMC12390311; doi:10.3390/toxics13080644)
Supplement: Supplementary file 1 [file toxics-13-00644-s001.zip › Supplementary Table S4.pdf]

Table S4 Contamination factor (CF) of potentially toxic elements in soil from all sampling locations in the Upper Ohře River Basin

	CF										
	**Al**	**As**	**Cd**	**Co**	**Cr**	**Cu**	**Fe**	**Mn**	**Ni**	**Pb**	**Zn**
**L1**	0.28	3.83	1.99	0.70	0.63	0.04	0.62	1.43	0.31	0.91	0.21
**L2**	0.58	5.10	12.36	1.66	2.77	0.78	1.27	3.43	1.24	3.11	0.66
**L3**	0.57	6.54	7.37	1.66	2.91	0.78	1.17	2.63	1.77	3.18	0.68
**L4**	0.39	4.69	2.07	1.05	1.78	0.48	0.87	1.56	0.94	2.88	0.32
**L5**	0.33	5.28	4.03	0.97	2.39	0.56	0.71	1.59	0.97	2.16	0.46
**L6**	0.61	10.43	5.28	1.38	3.55	0.96	1.17	2.61	1.42	4.07	0.59
**L7**	0.55	8.49	4.05	1.17	2.32	0.43	1.07	3.27	1.02	1.97	0.36
**L8**	0.42	6.45	2.15	0.88	3.71	0.25	0.79	2.19	0.76	1.30	0.24
**L9**	0.43	7.74	5.32	0.97	2.06	0.55	0.81	1.64	1.02	1.97	0.49
**L10**	0.35	6.67	2.85	0.91	1.79	0.34	0.75	1.49	0.72	1.77	0.29
**L11**	0.50	9.02	3.15	1.16	2.18	0.41	0.95	1.56	0.92	1.74	0.34
**L12**	0.54	14.26	6.48	1.40	2.53	0.54	1.20	1.52	1.22	3.26	0.56
**L13**	0.44	14.15	5.29	1.10	2.24	0.86	0.98	1.07	1.01	2.30	0.45
**L14**	0.59	11.56	3.67	1.66	2.89	0.57	1.35	2.23	1.40	2.25	0.45
**L15**	0.44	32.71	10.21	1.59	2.04	2.48	1.37	1.89	1.65	4.47	0.91
**L16**	0.50	39.76	12.73	1.95	2.67	4.14	1.39	1.99	2.18	9.35	1.33
**L17**	0.49	40.14	13.11	1.83	2.58	5.16	1.47	1.88	1.90	9.08	1.02
