# Supplementary material for: Assessment of Potentially Toxic Element Pollution in Surface Soils of the Upper Ohře River Basin
Source: Toxics. 2025 Jul 30;13(8):644. doi: 10.3390/toxics13080644 (PMC12390311; doi:10.3390/toxics13080644)
Supplement: Supplementary file 1 [file toxics-13-00644-s001.zip › Supplementary Table S5.pdf]

Table S5 Ecological risk factor (Er) of potentially toxic elements from all sampling locations in the Upper Ohře River Basin

	Er										
	**Al**	**As**	**Cd**	**Co**	**Cr**	**Cu**	**Fe**	**Mn**	**Ni**	**Pb**	**Zn**
**L1**	1.41	38.30	59.69	3.48	3.15	0.09	0.62	1.43	1.57	4.56	0.21
**L2**	2.89	51.01	370.66	8.31	13.86	1.56	1.27	3.43	6.19	15.53	0.66
**L3**	2.83	65.44	221.22	8.31	14.54	1.57	1.17	2.63	8.87	15.89	0.68
**L4**	1.96	46.90	62.10	5.23	8.89	0.95	0.87	1.56	4.68	14.42	0.32
**L5**	1.66	52.78	121.04	4.87	11.95	1.11	0.71	1.59	4.85	10.81	0.46
**L6**	3.03	104.34	158.27	6.90	17.75	1.92	1.17	2.61	7.09	20.35	0.59
**L7**	2.73	84.86	121.35	5.86	11.59	0.86	1.07	3.27	5.12	9.87	0.36
**L8**	2.12	64.55	64.47	4.40	18.56	0.50	0.79	2.19	3.82	6.51	0.24
**L9**	2.17	77.42	159.62	4.86	10.31	1.10	0.81	1.64	5.08	9.84	0.49
**L10**	1.75	66.74	85.64	4.56	8.96	0.68	0.75	1.49	3.60	8.86	0.29
**L11**	2.52	90.23	94.41	5.78	10.89	0.82	0.95	1.56	4.60	8.71	0.34
**L12**	2.72	142.63	194.49	7.01	12.63	1.08	1.20	1.52	6.12	16.28	0.56
**L13**	2.20	141.53	158.70	5.48	11.18	1.73	0.98	1.07	5.05	11.52	0.45
**L14**	2.97	115.58	110.01	8.28	14.44	1.13	1.35	2.23	7.00	11.26	0.45
**L15**	2.20	327.12	306.20	7.96	10.20	4.96	1.37	1.89	8.23	22.36	0.91
**L16**	2.50	397.55	382.00	9.77	13.34	8.27	1.39	1.99	10.90	46.73	1.33
**L17**	2.45	401.42	393.19	9.14	12.91	10.33	1.47	1.88	9.51	45.41	1.02
