# Supplementary material for: Assessment of Potentially Toxic Element Pollution in Surface Soils of the Upper Ohře River Basin
Source: Toxics. 2025 Jul 30;13(8):644. doi: 10.3390/toxics13080644 (PMC12390311; doi:10.3390/toxics13080644)
Supplement: Supplementary file 1 [file toxics-13-00644-s001.zip › Supplementary Table S6.pdf]

Table S6 Hazard quotient (HQ) of potentially toxic elements for children from all sampling locations in the Upper Ohře River Basin. Value of HQ < 1 indicates low risk of exposure

	HQ										
	**Al**	**As**	**Cd**	**Co**	**Cr**	**Cu**	**Fe**	**Mn**	**Ni**	**Pb**	**Zn**
**L1**	0.279	0.326	0.003	0.344	0.038	0.0005	0.348	0.069	0.0001	0.057	0.002
**L2**	0.573	0.435	0.016	0.822	0.169	0.009	0.718	0.165	0.0003	0.193	0.007
**L3**	0.561	0.558	0.010	0.821	0.177	0.009	0.662	0.127	0.0004	0.197	0.007
**L4**	0.387	0.400	0.003	0.517	0.108	0.005	0.492	0.075	0.0002	0.179	0.003
**L5**	0.328	0.450	0.005	0.482	0.146	0.006	0.399	0.076	0.0002	0.134	0.005
**L6**	0.600	0.889	0.007	0.682	0.216	0.011	0.663	0.125	0.0003	0.253	0.006
**L7**	0.540	0.723	0.005	0.580	0.141	0.005	0.602	0.157	0.0002	0.123	0.004
**L8**	0.420	0.550	0.003	0.435	0.226	0.003	0.446	0.105	0.0002	0.081	0.002
**L9**	0.429	0.660	0.007	0.481	0.126	0.006	0.458	0.079	0.0002	0.122	0.005
**L10**	0.346	0.569	0.004	0.451	0.109	0.004	0.422	0.072	0.0002	0.110	0.003
**L11**	0.498	0.769	0.004	0.572	0.133	0.005	0.537	0.075	0.0002	0.108	0.003
**L12**	0.538	1.216	0.008	0.693	0.154	0.006	0.679	0.073	0.0003	0.202	0.006
**L13**	0.436	1.206	0.007	0.541	0.136	0.010	0.551	0.052	0.0002	0.143	0.005
**L14**	0.588	0.985	0.005	0.819	0.176	0.006	0.764	0.107	0.0003	0.140	0.005
**L15**	0.436	2.788	0.013	0.787	0.124	0.028	0.775	0.091	0.0004	0.278	0.009
**L16**	0.496	3.389	0.017	0.966	0.163	0.046	0.782	0.096	0.0005	0.580	0.013
**L17**	0.486	3.422	0.017	0.904	0.157	0.058	0.832	0.091	0.0004	0.564	0.010
