# Supplementary material for: Assessment of Potentially Toxic Element Pollution in Surface Soils of the Upper Ohře River Basin
Source: Toxics. 2025 Jul 30;13(8):644. doi: 10.3390/toxics13080644 (PMC12390311; doi:10.3390/toxics13080644)
Supplement: Supplementary file 1 [file toxics-13-00644-s001.zip › Supplementary Table S7.pdf]

Table S7 Hazard quotient (HQ) of potentially toxic elements for adults from all sampling locations in the Upper Ohře River Basin. Value of HQ < 1 indicates low risk of exposure

	HQ										
	**Al**	**As**	**Cd**	**Co**	**Cr**	**Cu**	**Fe**	**Mn**	**Ni**	**Pb**	**Zn**
**L1**	0.021	0.009	0.0001	0.010	0.001	0.0000	0.027	0.005	0.0001	0.002	0.0002
**L2**	0.044	0.012	0.0005	0.023	0.005	0.0007	0.055	0.013	0.0004	0.005	0.0005
**L3**	0.043	0.016	0.0003	0.023	0.005	0.0007	0.051	0.010	0.0006	0.006	0.0005
**L4**	0.030	0.011	0.0001	0.015	0.003	0.0004	0.038	0.006	0.0003	0.005	0.0003
**L5**	0.025	0.013	0.0001	0.014	0.004	0.0005	0.031	0.006	0.0003	0.004	0.0004
**L6**	0.046	0.025	0.0002	0.019	0.006	0.0008	0.051	0.010	0.0005	0.007	0.0005
**L7**	0.041	0.021	0.0001	0.016	0.004	0.0004	0.046	0.012	0.0003	0.003	0.0003
**L8**	0.032	0.016	0.0001	0.012	0.006	0.0002	0.034	0.008	0.0003	0.002	0.0002
**L9**	0.033	0.019	0.0002	0.014	0.004	0.0005	0.035	0.006	0.0003	0.003	0.0004
**L10**	0.027	0.016	0.0001	0.013	0.003	0.0003	0.032	0.005	0.0002	0.003	0.0002
**L11**	0.038	0.022	0.0001	0.016	0.004	0.0004	0.041	0.006	0.0003	0.003	0.0003
**L12**	0.041	0.035	0.0002	0.020	0.004	0.0005	0.052	0.006	0.0004	0.006	0.0004
**L13**	0.033	0.034	0.0002	0.015	0.004	0.0007	0.042	0.004	0.0003	0.004	0.0003
**L14**	0.045	0.028	0.0001	0.023	0.005	0.0005	0.058	0.008	0.0005	0.004	0.0003
**L15**	0.033	0.079	0.0004	0.022	0.004	0.0021	0.059	0.007	0.0006	0.008	0.0007
**L16**	0.038	0.096	0.0005	0.027	0.005	0.0035	0.060	0.007	0.0007	0.016	0.0010
**L17**	0.037	0.097	0.0005	0.026	0.004	0.0044	0.064	0.007	0.0006	0.016	0.0008
