# Supplementary material for: Assessment of Potentially Toxic Element Pollution in Surface Soils of the Upper Ohře River Basin
Source: Toxics. 2025 Jul 30;13(8):644. doi: 10.3390/toxics13080644 (PMC12390311; doi:10.3390/toxics13080644)
Supplement: Supplementary file 1 [file toxics-13-00644-s001.zip › Supplementary Table S8.pdf]

**Table S8** Carcinogenic risk (CR) of potentially toxic elements for children from all sampling locations in the Upper Ohře River Basin. Value of CR exceeding $1 \times 10^{-4}$ indicate a high-risk level of cancer to human health

| | | | CR | | |
|---|---|---|---|---|---|
| | **As** | **Cd** | **Cr** | **Ni** | **Pb** |
| **L1** | 1.47E-04 | 9.86E-07 | 5.76E-05 | 2.34E-06 | 1.69E-06 |
| **L2** | 1.96E-04 | 6.12E-06 | 2.53E-04 | 9.19E-06 | 5.74E-06 |
| **L3** | 2.51E-04 | 3.65E-06 | 2.66E-04 | 1.32E-05 | 5.87E-06 |
| **L4** | 1.80E-04 | 1.03E-06 | 1.63E-04 | 6.95E-06 | 5.33E-06 |
| **L5** | 2.02E-04 | 2.00E-06 | 2.19E-04 | 7.20E-06 | 3.99E-06 |
| **L6** | 4.00E-04 | 2.61E-06 | 3.24E-04 | 1.05E-05 | 7.52E-06 |
| **L7** | 3.25E-04 | 2.00E-06 | 2.12E-04 | 7.61E-06 | 3.65E-06 |
| **L8** | 2.48E-04 | 1.06E-06 | 3.39E-04 | 5.67E-06 | 2.41E-06 |
| **L9** | 2.97E-04 | 2.64E-06 | 1.89E-04 | 7.55E-06 | 3.64E-06 |
| **L10** | 2.56E-04 | 1.41E-06 | 1.64E-04 | 5.34E-06 | 3.27E-06 |
| **L11** | 3.46E-04 | 1.56E-06 | 1.99E-04 | 6.83E-06 | 3.22E-06 |
| **L12** | 5.47E-04 | 3.21E-06 | 2.31E-04 | 9.09E-06 | 6.02E-06 |
| **L13** | 5.43E-04 | 2.62E-06 | 2.04E-04 | 7.49E-06 | 4.26E-06 |
| **L14** | 4.43E-04 | 1.82E-06 | 2.64E-04 | 1.04E-05 | 4.16E-06 |
| **L15** | 1.25E-03 | 5.06E-06 | 1.87E-04 | 1.22E-05 | 8.26E-06 |
| **L16** | 1.52E-03 | 6.31E-06 | 2.44E-04 | 1.62E-05 | 1.73E-05 |
| **L17** | 1.54E-03 | 6.50E-06 | 2.36E-04 | 1.41E-05 | 1.68E-05 |
