# Supplementary material for: Assessment of Potentially Toxic Element Pollution in Surface Soils of the Upper Ohře River Basin
Source: Toxics. 2025 Jul 30;13(8):644. doi: 10.3390/toxics13080644 (PMC12390311; doi:10.3390/toxics13080644)
Supplement: Supplementary file 1 [file toxics-13-00644-s001.zip › Supplementary Table S9.pdf]

Table S9 Carcinogenic risk (CR) of potentially toxic elements for adults from all sampling locations in the Upper Ohře River Basin. Value of CR exceeding 1×10^{-4} indicate a high-risk level of cancer to human health

	CR				
	**As**	**Cd**	**Cr**	**Ni**	**Pb**
**L1**	4.18E-06	2.80E-08	1.64E-06	3.62E-06	4.79E-08
**L2**	5.56E-06	1.74E-07	7.20E-06	1.42E-05	1.63E-07
**L3**	7.13E-06	1.04E-07	7.56E-06	2.04E-05	1.67E-07
**L4**	5.11E-06	2.92E-08	4.62E-06	1.08E-05	1.51E-07
**L5**	5.75E-06	5.68E-08	6.21E-06	1.11E-05	1.14E-07
**L6**	1.14E-05	7.43E-08	9.22E-06	1.63E-05	2.14E-07
**L7**	9.25E-06	5.70E-08	6.03E-06	1.18E-05	1.04E-07
**L8**	7.04E-06	3.03E-08	9.65E-06	8.77E-06	6.84E-08
**L9**	8.44E-06	7.49E-08	5.36E-06	1.17E-05	1.03E-07
**L10**	7.28E-06	4.02E-08	4.66E-06	8.26E-06	9.30E-08
**L11**	9.84E-06	4.43E-08	5.66E-06	1.06E-05	9.15E-08
**L12**	1.56E-05	9.13E-08	6.56E-06	1.41E-05	1.71E-07
**L13**	1.54E-05	7.45E-08	5.81E-06	1.16E-05	1.21E-07
**L14**	1.26E-05	5.17E-08	7.51E-06	1.61E-05	1.18E-07
**L15**	3.57E-05	1.44E-07	5.30E-06	1.89E-05	2.35E-07
**L16**	4.33E-05	1.79E-07	6.93E-06	2.51E-05	4.91E-07
**L17**	4.38E-05	1.85E-07	6.71E-06	2.19E-05	4.77E-07
